# Constrictive Pericarditis Mimicking Heart Failure: Hemodynamic Catheterization as the Diagnostic Key

**DOI:** 10.7759/cureus.106747

**Published:** 2026-04-09

**Authors:** Sina Bakhshaei, Benjamin Szumowski, Rastina Mehrani, Collin Jacobsen, Sinan Sarsam

**Affiliations:** 1 Cardiovascular Disease, Temecula Valley Hospital, Temecula, USA; 2 Cardiovascular Disease, Southwest Healthcare Medical Education Consortium, Temecula, USA; 3 Internal Medicine, Southwest Healthcare Medical Education Consortium, Temecula, USA; 4 Cardiovascular Disease, School of Medicine, St. George's University, Temecula, USA; 5 School of Medicine, University of California Riverside School of Medicine, Riverside, USA

**Keywords:** chronic constrictive pericarditis, hemodynamic, left/right heart catheterization, pericardiectomy, square root sign, ventricular interdependence

## Abstract

Constrictive pericarditis (CP) is an uncommon but potentially reversible cause of heart failure, characterized by pericardial thickening, fibrosis, and loss of elasticity. Early recognition is essential because timely intervention can be curative. We report the case of a 64-year-old man with multiple comorbidities and recurrent heart failure admissions who was ultimately diagnosed with CP. Echocardiography suggested constrictive physiology (including annulus reversus), and hemodynamic catheterization demonstrated elevated right atrial pressure with rapid “y” descent, equalization of diastolic pressures across chambers, and a classic square root sign. He underwent pericardiectomy with an uneventful recovery and marked symptomatic improvement. This case highlights the diagnostic complexity of CP and underscores the central role of invasive hemodynamics in distinguishing it from other causes of heart failure, including restrictive cardiomyopathy and diastolic heart failure.

## Introduction

Constrictive pericarditis (CP) is the end-stage manifestation of chronic pericardial inflammation, characterized by progressive fibrosis, scarring, and often calcification of the visceral and parietal layers [1,2]. Loss of pericardial compliance restricts diastolic filling, reduces cardiac output, and produces signs of right-sided heart failure. Clinically, this results in a constellation of findings including elevated jugular venous pressure, peripheral edema, ascites, and exercise intolerance, which often overlap with other causes of heart failure.

Although relatively uncommon, CP occurs in approximately 1.8% of patients following acute pericarditis and in fewer than 0.5% after cardiac surgery [3]. Reported etiologies include idiopathic or viral pericarditis, prior cardiac surgery, chest radiation, autoimmune conditions (e.g., rheumatoid arthritis, systemic lupus erythematosus), infections such as tuberculosis, trauma, and malignancy [1,4,5]. However, in a substantial proportion of cases, no clear etiology is identified, and the condition is classified as idiopathic.

Despite advances in multimodality imaging, CP can closely mimic restrictive cardiomyopathy. In equivocal cases, invasive hemodynamic assessment remains the gold standard for diagnosis [1,2]. This diagnostic overlap may lead to delayed or missed diagnosis, particularly in patients with recurrent heart failure admissions and nonspecific clinical findings.

In this report, we present a case of CP initially presenting as recurrent decompensated heart failure with atypical features, including clinical signs of volume overload and elevated B-type natriuretic peptide (BNP), highlighting the importance of maintaining a high index of suspicion and the critical role of invasive hemodynamic assessment in establishing the diagnosis.

## Case presentation

A 64-year-old male with type 2 diabetes mellitus, chronic kidney disease stage 2, dyslipidemia, and heart failure with preserved ejection fraction (HFpEF) presented with progressive dyspnea. He had multiple recent hospital admissions for decompensated heart failure. These recurrent presentations were previously attributed to HFpEF without a definitive alternative diagnosis.

On arrival, his vital signs were notable for a heart rate of 121 bpm and blood pressure of 99/68 mmHg. Physical examination revealed an irregularly irregular rhythm with tachycardia, anasarca, and jugular venous distention to 10 cm, with a positive Kussmaul sign, although lung fields were clear bilaterally. Electrocardiography demonstrated atrial fibrillation with rapid ventricular response. Serial high-sensitivity troponins were within normal limits, but B-type natriuretic peptide (BNP) was elevated at 1707 pg/mL. The atrial fibrillation was a new diagnosis at the time of presentation.

Bedside point-of-care ultrasound (POCUS) demonstrated preserved left ventricular ejection fraction (LVEF), no evidence of tamponade or pulmonary edema, but a dilated (2.3 cm) and non-collapsible inferior vena cava (IVC). In the emergency department, intravenous diltiazem (10 mg) effectively reduced the heart rate, and the patient was subsequently started on an amiodarone infusion as part of a rate and rhythm control strategy. 

**Figure 1:**
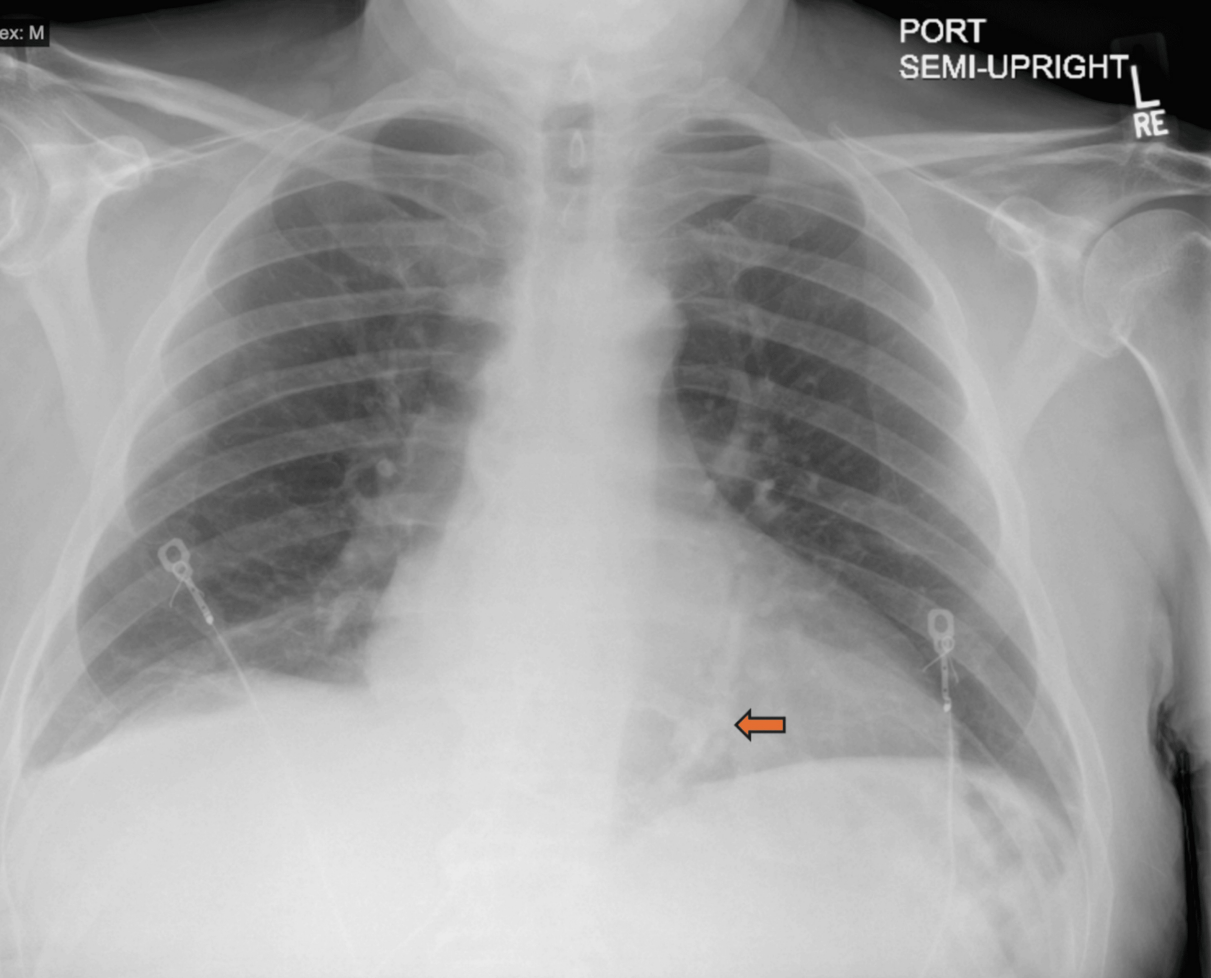
Chest X-ray showing curvilinear pericardial calcifications (arrow).

**Figure 2:**
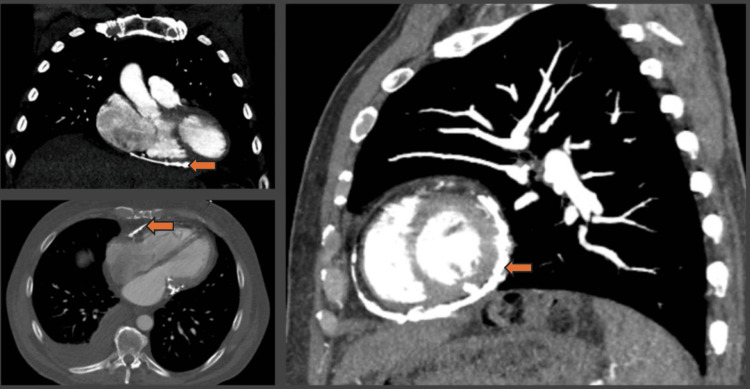
Chest CT demonstrating extensive pericardial calcifications (arrows), raising clinical suspicion for CP. CT: computed tomography; CP: constrictive pericarditis.

**Figure 3:**
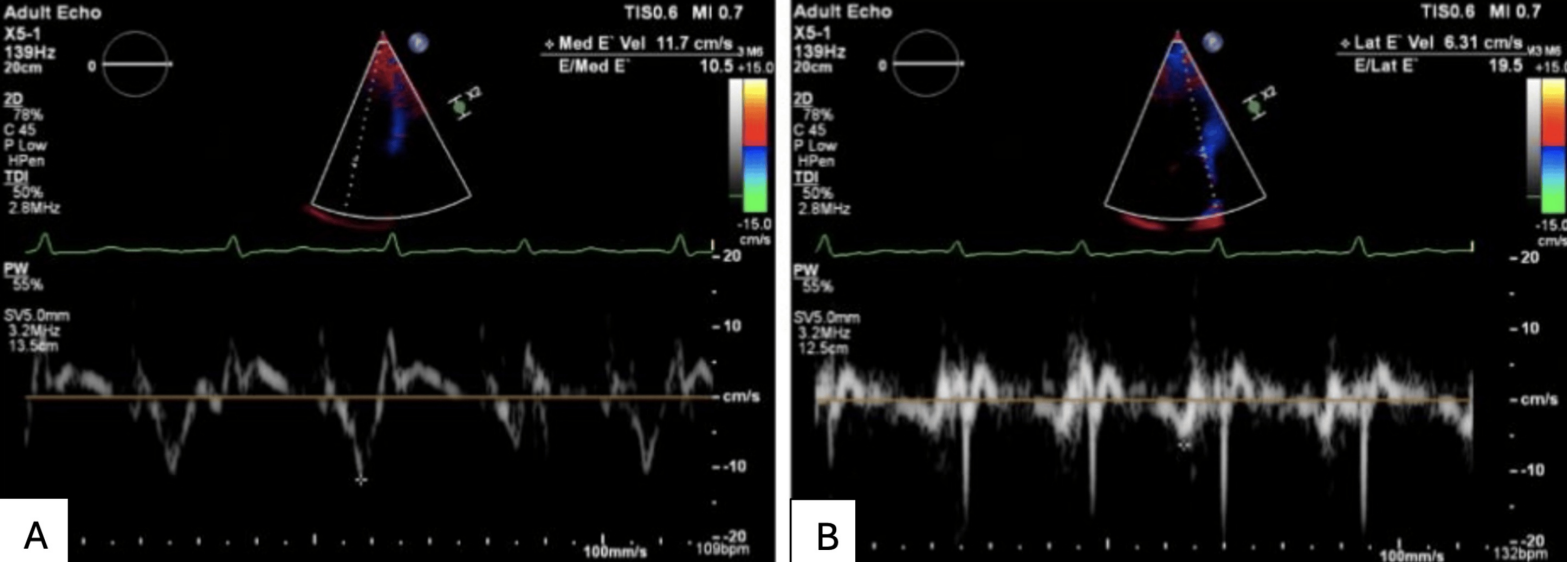
Transthoracic echocardiogram (apical four-chamber view) showing tissue Doppler imaging of medial (A) and lateral (B) mitral annular early diastolic velocities (e′), demonstrating annulus reversus (medial e′ > lateral e′), consistent with constrictive pericardial physiology.

Chest X-ray revealed no acute cardiopulmonary abnormalities but was suggestive of pericardial calcifications (Figure 1). Subsequently, computed tomography (CT) of the chest confirmed extensive pericardial calcifications (Figure 2). No evidence of active infection, malignancy, or other secondary causes was identified on imaging. Transthoracic echocardiography (TTE) demonstrated preserved LVEF (55-60%), annulus reversus (Figure 3), and ventricular interdependence, findings consistent with constrictive physiology. Additional supportive findings included septal bounce and a plethoric inferior vena cava. Given the diagnostic uncertainty and to confirm constrictive physiology, the patient underwent transesophageal echocardiography followed by direct current cardioversion to restore sinus rhythm prior to simultaneous right and left heart catheterization.

**Figure 4:**
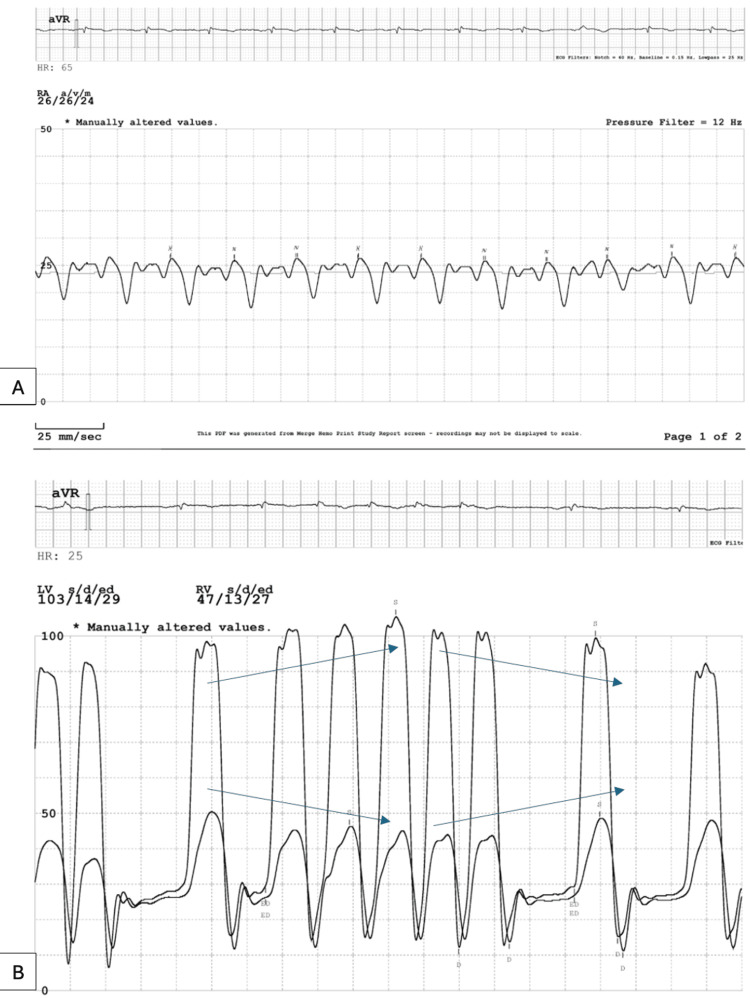
Invasive hemodynamics. (A) Right heart catheterization showing elevated right atrial pressures with prominent “x” and rapid “y” descents, equalization of diastolic pressures across chambers, and the classic “square root sign.” (B) Simultaneous right- and left-heart catheterization demonstrating respirophasic ventricular interdependence (arrows), a hallmark finding of CP. CP: constrictive pericarditis.

Right heart catheterization demonstrated elevated right atrial pressures (24 mmHg) with prominent “x” and rapid “y” descents, equalization of diastolic pressures across all cardiac chambers (24-30 mmHg), and the characteristic “square root sign” (Figure 4A). Furthermore, simultaneous right and left heart catheterization revealed respirophasic ventricular interdependence between the left and right ventricles (Figure 4B). Collectively, these hemodynamic findings confirmed the diagnosis of CP.

**Figure 5:**
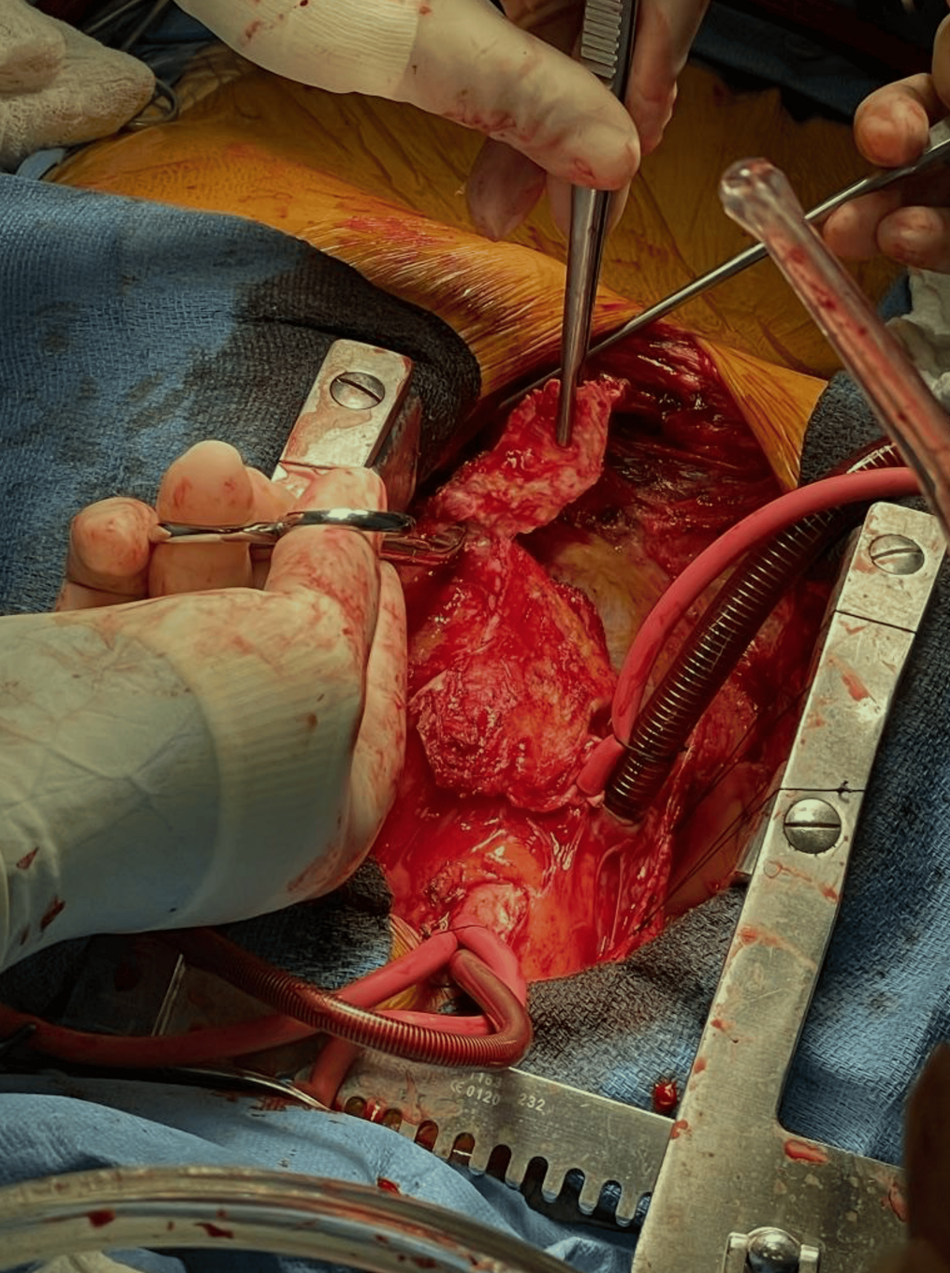
Intraoperative image of pericardiectomy Resected thickened and calcified pericardium.

The patient was initially started on gentle diuresis and close monitoring. Despite medical management, he continued to have signs of volume overload and elevated filling pressures. Given persistent hemodynamic compromise, cardiothoracic surgery was consulted, and pericardiectomy was planned. The patient subsequently underwent surgical pericardiectomy (Figure 5) without intraoperative complications.

Postoperatively, his recovery was uneventful, with marked improvement in dyspnea and functional status. After discharge, the patient continued recovery with participation in physical therapy and cardiac rehabilitation.

At three-month follow-up, the patient reported significant improvement in functional capacity, with resolution of dyspnea and no further heart failure-related hospitalizations. Physical examination demonstrated resolution of peripheral edema and normalization of jugular venous pressure. He remained in sinus rhythm without recurrence of atrial fibrillation. Repeat echocardiography showed improvement in filling dynamics and reduction in inferior vena cava congestion. Overall, the patient had a marked clinical recovery following pericardiectomy.

## Discussion

CP is an uncommon but clinically significant cause of heart failure that often masquerades as other cardiac conditions. Patients typically present with systemic venous congestion, including jugular venous distention, hepatomegaly, ascites, and peripheral edema. Low cardiac output may contribute to exertional dyspnea, fatigue, and tachycardia. Because these manifestations overlap with those of cardiac tamponade, restrictive cardiomyopathy, or decompensated HFpEF, the diagnosis is frequently delayed. Several published case series have similarly highlighted delays in diagnosis due to nonspecific presentations and overlap with HFpEF, particularly in patients with recurrent hospitalizations for decompensated heart failure [6].

The etiology of CP varies by region. In developed countries, most cases are idiopathic or post-viral in origin, whereas tuberculosis remains the predominant cause globally [7]. Other well-described etiologies include prior cardiac surgery, chest radiation, and autoimmune diseases such as rheumatoid arthritis and systemic lupus erythematosus [8]. Less commonly, CP may arise from malignancy, trauma, or uremia. Epidemiologic data from the United States suggest a prevalence of approximately 9-10 cases per million population between 2005 and 2014 [9], underscoring its rarity but clinical relevance. In our patient, an extensive evaluation for secondary causes (including history of prior pericarditis, cardiothoracic surgery, chest radiation, infectious etiologies, autoimmune conditions, and malignancy) did not reveal an identifiable cause, and the condition was therefore classified as idiopathic.

Diagnostic evaluation requires a multimodality approach. Echocardiography provides important early clues, such as annulus reversus, septal bounce, and exaggerated ventricular interdependence. Cross-sectional imaging with CT or MRI can demonstrate pericardial thickening or calcification. Nevertheless, in ambiguous cases, invasive hemodynamic catheterization remains the diagnostic gold standard. Characteristic findings include equalization of diastolic pressures across all cardiac chambers, a rapid “y” descent, the square root sign, and discordant left and right ventricular pressure changes with respiration [1,2]. Our patient exhibited each of these hemodynamic hallmarks, which confirmed the diagnosis and guided definitive therapy. Notably, despite these classic findings, the presence of elevated BNP and concomitant atrial fibrillation initially contributed to diagnostic uncertainty, as these features are more commonly associated with other forms of heart failure.

Management of CP depends on the stage of the disease. In early or transient forms, anti-inflammatory therapies, such as nonsteroidal anti-inflammatory drugs, colchicine, or corticosteroids, may lead to resolution [10]. Diuretics can provide symptomatic relief by controlling volume status, but they are not curative. In chronic disease, complete pericardiectomy remains the only definitive treatment. Surgical intervention has been shown to significantly improve symptoms and long-term survival when performed before the development of advanced hepatic dysfunction or myocardial atrophy [1,10]. In the present case, pericardiectomy resulted in an excellent clinical outcome, with marked improvement in functional capacity and stable hemodynamics at follow-up. 

This case underscores the diagnostic challenges of CP in patients with multiple comorbidities and recurrent heart failure admissions. It highlights the pivotal role of invasive hemodynamic assessment in distinguishing CP from restrictive cardiomyopathy and other mimicking conditions. Early recognition and timely referral for surgical intervention can result in substantial improvements in functional status and overall prognosis. Clinicians should maintain a high index of suspicion for constrictive physiology in patients with recurrent heart failure symptoms despite preserved ejection fraction, particularly when clinical features are atypical or disproportionate to the presumed diagnosis.

## Conclusions

CP is a rare but surgically treatable cause of heart failure. While it classically presents with systemic venous congestion and features of right-sided heart failure, advanced disease may also manifest with left-sided symptoms, including pulmonary congestion and reduced exercise tolerance. Because its clinical presentation can closely mimic other cardiac conditions, timely recognition requires a multimodal diagnostic approach, with invasive hemodynamic catheterization remaining the gold standard for confirmation. Early identification and prompt referral for pericardiectomy are essential, as surgical intervention can lead to substantial symptomatic improvement and favorable long-term outcomes. This case highlights the importance of maintaining a high index of suspicion in patients with recurrent decompensated heart failure and underscores the critical role of hemodynamic assessment in distinguishing CP from other causes of diastolic dysfunction.
